# Predictive Value of Galectin-3 and Brachial-Ankle Pulse Wave Velocity for Coronary Artery Calcification in Coronary Arteriography Patients

**DOI:** 10.1155/2022/1865736

**Published:** 2022-05-16

**Authors:** Lei Tian, Fenghua Ding, Ruiyan Zhang

**Affiliations:** Department of Cardiovascular Medicine, Ruijin Hospital, Shanghai Jiao Tong University School of Medicine, Shanghai, China

## Abstract

**Objectives:**

To study the predictive value for coronary artery calcification (CAC) of plasma galectin-3 and brachial-ankle pulse wave velocity (BaPWV) in coronary arteriography (CAG) patients.

**Methods:**

Patients who received coronary arteriography (CAG) examination were recruited. The level of plasma galectin-3 was measured by the enzyme-linked immunosorbent assay. The arterial stiffness was analyzed by BaPWV and ankle-brachial index (ABI) which were measured using a volume-plethysmographic device. Receiver operating characteristic (ROC) curve was used to analyze the prognostic value of galectin-3 or BaPWV for coronary artery calcification (CAC).

**Results:**

The level of galectin-3 and BaPWV was significantly higher in CAC patients compared with that in control (*p* < 0.01). The level of plasma galectin-3 was positively correlated with BaPWV (*r* = −0.296, *p* < 0.01) and negatively correlated with ABI (*r* = −0.296, *p* < 0.01). ROC curve analysis revealed that galectin-3 ≥5.90 ng/ml was the most powerful predictor for CAC with sensitivity of 85.5% and specificity of 83.5%. The area under the curve (AUC) was 0.916. When the level of BaPWV was more than 1909 m/s, the sensitivity and specificity were 61.8% and 69.6%, respectively, for predicting CAC. The AUC was 0.646.

**Conclusions:**

The level of plasma galectin-3 increases significantly in CAC patients compared to control, and its level is related to BaPWV and ABI. Galectin-3 and BaPWV can be used to predict CAC, and the diagnosis value (sensitivity and specificity) of galectin-3 for CAC is better than that of BaPWV.

## 1. Background

Arterial calcification is an unfavorable event that predicts cardiovascular morbidity and mortality. Coronary calcium has been proved to predict coronary events in a population-based sample [1]. Besides, Kramer et al. found that coronary artery calcification (CAC) is strongly associated with diabetes and chronic kidney disease [2]. A combination of organic and functional stiffness of the arterial wall can be reflected by arterial stiffness [3]. Large artery stiffness is a high-risk factor in cardiovascular pathological processes including increasing cardiac workload and reducing coronary flow, leading to left ventricular hypertrophy, coronary ischemia, and heart failure [4].

Pulse wave velocity is the reliable method to assess arterial stiffness. Carotid femoral pulse wave velocity (cfPWV) is the most validated technique and is regarded as the gold standard for PWV measurement [5]; however, cfPWV is not widely implemented because of its inconvenience associated with the measuring devices. In contrast, BaPWV, an easy and reproducible measurement, has been widely and rapidly spread in regular clinical and epidemiological settings [6]. A 1 m/s increase in BaPWV has been proved to be associated with 12% increase in the risk of cardiovascular events in the general population with hypertension, diabetes, or end-stage renal disease, and other high-risk individuals [4].

The role of galectin-3 in atherosclerosis is widely observed, but the conclusion is not always consistent. Endothelial cells, macrophages, and vascular smooth muscle cells (VSMCs) are three most important cells in the process of atherosclerosis pathology, and galectin-3 affects all these kinds of cells [7]. Galectin-3 aggravates oxLDL-mediated endothelial injury, promotes endocytosis of lipoprotein in macrophage, and stimulates VSMCs proliferation and migration [8, 9]. However, Iacobini et al. found that the atherosclerosis plaque area in galectin-3-knockout mice is much higher than that of wild-type mice [10]. The macrophage infiltration, accumulation of oxidized low-density lipoprotein, and markers of systemic inflammation increased in galectin-3-knockout mice compared with the wild type [8, 10, 11].

Coronary arteriography (CAG) is regarded as a gold standard method in the diagnosis of coronary artery disease. In our clinical practices, we need to evaluate coronary calcification and then decide whether to treat atherosclerotic plaque during CAG examination. The atherosclerotic plaque extent largely affects the follow-up treatment strategy during CAG process and predicts the major adverse cardiovascular events (MACEs) in acute coronary syndrome (ACS) patients [12]. The purpose of this research was to investigate the correlation between galectin-3, BaPWV, and CAC and verify the hypothesis that an elevated serum galectin-3 level is a useful biomarker for CAC in CAG patients.

## 2. Material and Methods

### 2.1. Selection of the Study Population

The study group consecutive recruited 134 patients who underwent invasive procedures—coronary angiography and angioplasty at the Cardiology Department. The exclusion criteria were having prior HF, severe valvular heart diseases, acute renal failure, hyperthyroidism, coexisting cancers, acute muscle injury and myopathy, connective tissue diseases and cirrhosis. White blood cell (WBC) counts and biochemical measurements were performed with standard laboratory methods. Demographic characteristics, clinical variables and patient history were obtained from patient interviews and medical records.

### 2.2. Ethics Statement

The research protocol conforms to the ethical guidelines of the 1975 Declaration of Helsinki as reflected in a priori approval by local medical ethics committee. All patients provided written informed consent documents.

### 2.3. Study Design

We defined CAC as “readily apparent densities seen within the artery wall and site of lesion as an X-ray-absorbing mass” according to the previous method [13], and CAC was classified as none or mild, moderate (densities noted only during the cardiac cycle before contrast injection), or severe (densities noted without cardiac motion before contrast injection generally involving both sides of the arterial wall). Lesions were divided into Calc lesions (moderate or severe calcification; 55 lesions) and non-Calc lesions (none or mild calcification; 79 lesions), based on the lesion calcium definition. Two authors independently assessed the severity of calcification. All disagreements were settled by discussion or consulted a third author until a consensus was achieved.

### 2.4. Measurement of Galectin-3 and Arterial Stiffness

12-hour fasting venous blood samples of patients were withdrawn and collected in ethylenediamine tetraacetic acid (EDTA). The blood samples were centrifuged at 1200 g for 15 min at 4°C. The plasma was then extracted and stored at −80°C. Plasma galectin-3 levels were measured in duplicate with an enzyme-linked immunosorbent assay (ELISA) (BG Medicine, Waltham, MA, USA). BaPWV and ABI were measured using a volume-plethysmographic device (BP-203RPEII, Nihon Colin, Japan) as previously reported [14, 15]. We used the average value of BaPWV and ABI values on both sides for analysis.

### 2.5. Statistical Analysis

The data were presented as mean ± standard deviation (SD) and analyzed by using the IBM SPSS 22.0 software package. Difference between two groups was compared by *t*-test. A *p* < 0.05 was considered statistically significant. Association of two sets of data was performed with Pearson correlation analysis. The ROC curve was used to analyze the predictive value of galectin-3 or BaPWV for CAC.

## 3. Results

### 3.1. Baseline Characteristics

From 135 patients who received CAG examination, 55 had calcification, 79 without calcification. The patients with calcification and noncalcification showed no significant differences with respect to age, gender, body mass index (BMI), total cholesterol (TC), low-density lipoprotein (LDL), high-density lipoprotein (HDL), white blood cell (WBC) count, creatinine, and uric acid (*p* > 0.05Table 1). There were no statistical differences for a history of hypertension, diabetes, current smoking, and cerebral infarction between calcification and noncalcification patients.

### 3.2. Comparison of ABI, BaPWV, and Galectin-3 in Calcification and Noncalcification Patients

**Figure 1:**
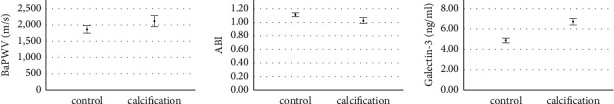
Comparison of the level of galectin-3, BaPWV, and ABI in CAC and control patients. Galectin-3, BaPWV, and ABI were significantly different in CAC patients compared with that in control (*p* < 0.01).

Figure 1 shows the comparisons of BaPWV, ABI, and galectin-3 between calcification and noncalcification patients. The level of BaPWV was statistically higher in the calcification group compared with that in noncalcification group (2114.24 ± 620.72 m/s vs 1861.23 ± 528.61 m/s, *p* < 0.01, Figure 1). The mean ABI value was 1.02 ± 0.16 in calcification patients, which was significantly lower than that in noncalcification patients (1.11 ± 0.17, *p* < 0.01). Meanwhile, the higher level of galectin-3 in calcification (6.71 ± 1.18 ng/ml) in contrast to noncalcification (4.88 ± 1.01 ng/ml) was significant.

### 3.3. Correlations of Serum Galectin-3 Level with BaPWV and ABI

**Figure 2:**
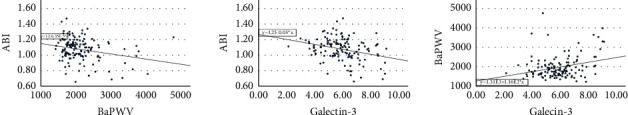
Correlation analysis between galectin-3 level and arterial stiffness parameters (BaPWV and ABI). Galectin-3 level was positively correlated with BaPWV (*r* = 0.282, *p* < 0.01) but negatively correlated with ABI (*r* = −0.296, *p* < 0.01). The level of BaPWV was negatively correlated with ABI (*r* = −0.252, *p* < 0.01).

Pearson correlation analysis showed that the level of ABI was negatively correlated with BaPWV (*r* = −0.252, *p* < 0.01, Figure 2). The correlation between the circulating galectin-3 level and large arteries stiffness was also assessed. The results of the present study revealed that the level of plasma galectin-3 was negatively correlated with ABI (*r* = −0.296, *p* < 0.01, Figure 2), and the positive correlation between plasma galectin-3 and BaPWV was also significant (*r* = 0.282, *p* < 0.01, Figure 2).

### 3.4. Analysis of the Diagnostic Value of Plasma Galectin-3 and BaPWV for Calcification with ROC Curve

**Figure 3:**
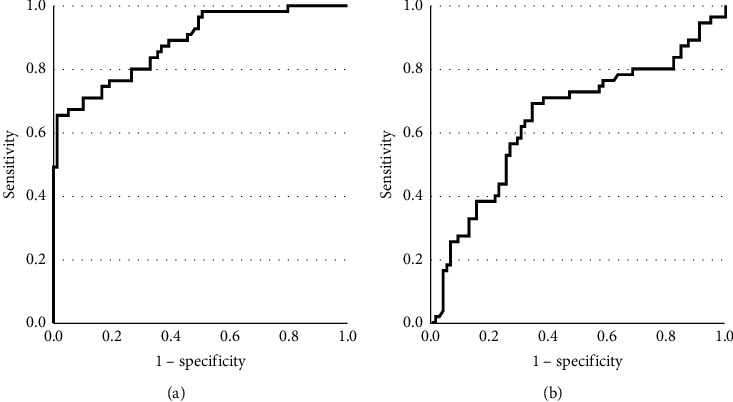
Analysis of the diagnostic value of galectin-3 and BaPWV for CAC with ROC curve. (a) ROC plot of galectin-3 level predicting CAC. The AUC was 0.916. When the cut-off value of galectin-3 was ≥5.90 ng/ml, the sensitivity and specificity were 83.5% and 85.5%, respectively, for predicting CAC. (b) ROC plot of BaPWV level predicting CAC. The AUC was 0.646. When the cut-off value of BaPWV was ≥1909 m/s, the sensitivity and specificity were 61.8% and 69.6%, respectively, for predicting CAC.

Receiver operating characteristic (ROC) curve was plotted according to the data of 55 calcification patients and 79 noncalcification patients. ROC curve analysis revealed that galectin-3 ≥5.90 ng/ml was the most powerful predictor for CAC with sensitivity of 85.5% and specificity of 83.5%. The area under the curve (AUC) was 0.916 (Figure 3(a)). Similarly, to elucidate whether BaPWV was also a powerful predictor of CAC, ROC curve analysis was performed. The AUC was 0.646. When the level of BaPWV was more than 1909 m/s, the sensitivity and specificity were 61.8% and 69.6%, respectively, for predicting CAC (Figure 3(b)).

## 4. Discussion

Arterial stiffness is complicate pathological processes which is related to a series of mechanisms, including arterial fibrosis, intimal thickened, and VSMCs phenotype transformation. BaPWV can be used to evaluate arterial stiffness in the previous studies [16, 17]. In the present study, we found that galectin-3 and BaPWV were significantly correlated, and they effectively predicted for CAC in patients who received CAG examination.

Galectin-3, a *β*-galactoside-binding lectin protein, participates in inflammatory and fibrotic processes. It is well-known that galectin-3 plays an important role in atherosclerosis development (the dark and bright of calcification). Galectin-3 was not only upregulated in vascular intimal calcification but also induces VSMCs migration and vascular intimal calcification [18, 19]. More importantly, galectin-3 day-to-day variation in serum is 3%, and there is no circadian variation of galectin-3 in serum [20]. The predictive value of galectin-3 for cardiovascular mortality and all-cause mortality in heart failure patients are well established [21]. A high concentration of galectin-3 was observed in coronary heart diseases and MI patients [22–24]. Whereas the important role of galectin-3 in arterial calcification, our previous meta-analysis has reported that galectin-3 is likely a predictor for the adverse outcomes in MI patients [25]. Since CAC is a reflection of severity of coronary heart diseases [26, 27], we evaluate the predictive value of galectin-3 on CAC. Our results indicated that the galectin-3 level was much higher in the calcification group than the noncalcification group, meanwhile, galectin-3 is an effective biomarker for CAC.

CAC is a well-known phenomenon in atherosclerosis. Nowadays, even though the drug-eluting stent is widely used to perform target lesion revascularization in the moderately or severely calcified lesions, total MACE in Calc patients was significantly higher than in non-Calc patients [13]. One of the major concerns of our study was the association of BaPWV with CAC. BaPWV has been regarded as a significant predictor of total cardiovascular events, cardiovascular mortality, and all-cause mortality [28]. In fact, functional alteration of the arteries also suggests the arterial impairment long before the appearance of clinic al lesions. In the population-based cohort studies, BaPWV increase significantly predicts total and cardiovascular mortality rates [29, 30]. The correlation of BaPWV to MACE in hemodialysis patients has also been reported [31]. It was our interests to investigate the association of BaPWV with CAC. We found that BaPWV was not only an independent risk factor of severe CAC but also had a positive correlation with galectin-3 in CAG patients.

Patients with calcified lesions are usually associated with higher MACE rate [13]. Although computed-tomography scan (CT) is a more reliable method to evaluate the coronary artery calcification (CAC), in the real world, a lot of coronary heart disease patients received the coronary angiography directly instead of CT. Coronary angiography can also be used to evaluate the level of coronary artery calcification, more importantly, target lesion revascularization (TLR) strategy is decided according to the Calc lesions conditions during coronary angiography [32]. Quantitative calcification of coronary lesions is associated with target lesion revascularization and binary restenosis rates in sirolimus-eluting stent treated patients [13]. Whereas the important value and widely used of CAG in clinical routine practices, we used CAG instead of CT to assess CAC in our research.

In terms of the important roles of BaPWV and galectin-3 in cardiovascular diseases, we observed them in CAG patients. The level of plasma galectin-3 increases significantly in CAC patients compared to control. Its level is related to BaPWV and ABI, indicating that galectin-3 may have been involved in the process of arterial calcification. BaPWV and galectin-3 can be used to predict CAC, and the diagnosis value (sensitivity and specificity) of galectin-3 for CAC is better than that of BaPWV.

## Figures and Tables

**Table 1:** Comparisons of clinical characteristics in CAC and control participants.

Characteristic	Calcification (55)	Non-calcification (79)	*p*-value
Age (y)	67.58 ± 8.92	67.80 ± 8.92	0.398
Male, *n*(%)	22(40.00)	33(41.80)	0.728
BMI (kg/m^2^)	23.42 ± 2.50	24.8 ± 3.94	0.181
HDL (mmol/L)	1.08 ± 0.25	1.10 ± 0.34	0.718
LDL (mmol/L)	2.61 ± 1.12	2.63 ± 0.76	0.796
WBC count (×10^5^)	6.39 ± 1.95	6.23 ± 1.71	0.166
Creatinine (*μ*mol/L)	82.60 ± 11.60	82.02 ± 18.57	0.046
Hypertension, *n* (%)	29(52.7)	31(39.2)	0.122
Diabetes, *n* (%)	9(16.4)	16(20.3)	0.570
Cerebral infarction, *n* (%)	10(18.2)	10(12.7)	0.377
Current smoking, *n* (%)	5(9.1)	8(10.1)	0.225
Galectin-3 (ng/ml)	6.72 ± 1.18	4.87 ± 1.01	<0.01
ABI	1.02 ± 0.16	1.11 ± 0.13	<0.01
BaPWV (m/s)	2114.24 ± 620.72	1861.23 ± 528.61	<0.01

The data are mean ± SD or numbers (percentages). BMI: body mass index; HDL: high-density lipoprotein; LDL: low-density lipoprotein; WBC: white blood cell; ABI: ankle-brachial index; BaPWV: brachial-ankle pulse wave velocity.

## Data Availability

The data presented in this study are available on reasonable request from the corresponding author.
